# Use of traditional medicines to cope with climate-sensitive diseases in a resource poor setting in Bangladesh

**DOI:** 10.1186/1471-2458-14-202

**Published:** 2014-02-25

**Authors:** Md Aminul Haque, Valérie R Louis, Revati Phalkey, Rainer Sauerborn

**Affiliations:** 1 Institute of Public Health, Heidelberg University, Im Neuenheimer Feld 324, 69120 Heidelberg, Germany; 2 Department of Population Sciences, University of Dhaka, Dhaka 1000, Bangladesh

**Keywords:** Traditional medicine, Health coping, Climate change, Resource-poor setting in Bangladesh

## Abstract

**Background:**

This study aims to explore the use of traditional medicines to cope with climate sensitive diseases in areas vulnerable to climate change. We assessed the extent to which traditional or alternative medicines were used for the treatment of the climate sensitive diseases by villagers as part of their health-coping strategies.

**Methods:**

The study deployed a mixed-method research design to know the health-coping strategies of the people in a resource-poor setting.

A cross sectional study was conducted from September 2010 to March 2011 among 450 households selected randomly in the districts of Rajshahi and Khulna, Bangladesh. The elder males or females of each household were interviewed. For qualitative methods, twelve focus group discussions (six with females and six with males) and fifteen key informant interviews were conducted by the research team, using interview guidelines on the use of traditional medicine.

**Results:**

Univariate analysis showed that the use of traditional medicines has increased among community members of all socio-economic and demographic backgrounds. Due to the increased incidence of disease and sickness respondents had to increase the use of their cultural means to cope with adverse health situations.

**Conclusions:**

A systematic collection of knowledge on the use of traditional medicines to cope with climate-sensitive diseases can help the adaptation of communities vulnerable to climate change. In addition it can be instrumental in creating a directory of traditional medicine components used for specific diseases and highlight the effectiveness and relevance of traditional medicines as health-coping strategies. This may be useful for policymakers, researchers, and development partners to adapt existing health care policy in resource-limited contexts. It may also encourage WHO, national and international institutions, such as pharmaceutical companies, to carry out research investigating the effectiveness of these traditional medicines and integrate them with modern medicine. Overall, it could increase the health coping capacity of people in a resource-poor setting and contribute to their adaptation capabilities.

## Background

Due to its geographic location, high population density, large proportion of poor population whose livelihood depends primarily on agriculture Bangladesh has become one of countries in the world to most vulnerable climate change [1-3]. The situation is going to worsen because climate change is projected to cause more extreme situations in Bangladesh, causing erratic and irregular patterns of droughts, flood and tropical cyclones [3]. All these will directly or indirectly impact negatively on people’s lives [4-8]. While the impact of climate change will effect on everyone’s lives in Bangladesh, the severity of the problems and the type of suffering will be unequally shared and distributed [9,10]. It will affect people differently according to their age, gender, location, occupation and social class [9,10]. Primarily, it will affect the poor and marginalized members of the society who do not have adequate access to resources. Given high level of poverty of Bangladesh, the problems associated with climate change will be widespread.

One of the core problems in both developed and developing countries is the consistent influence of climate change on the fundamental determinants of health [9]. According to the WHO report on the estimated global burden of diseases, different regions of the developed and developing world will experience different levels of exposure to diseases [11]. World Health Organization has grouped Bangladesh within the South East Asian Region ‘D’ sub-region, where mortality and disability adjusted to life-year’s are the highest in the world. Malaria and dengue diseases are projected to increase by 2.2 percent in Bangladesh due to climate change [12]. So the impending effect of climate change on human health in Bangladesh is expected to be high.

As a developing country, Bangladesh’s population is burdened by different types of diseases [13,14]. Bangladesh is also a disaster prone country. At least 174 natural disasters affected Bangladesh from 1974 to 2003 [15]. Extreme events such as floods, droughts and cyclones directly and indirectly affect the health of people of this country almost every year. For example, the total deaths caused by the flood in 2004 were about 800, and the cyclone of 1991 killed 1,38,000 people [16]. The projected effect of climate change will most likely make the disaster prone country more vulnerable, with high human health causalities.

Considering the socio-economic and demographic conditions of the country, vulnerability to infectious, water-borne, vector-borne and other types of communicable and non-communicable diseases is common [17]. The WHO estimated an increased incidence of malaria (from 1,556 in 1971 to 42,012 in 2004) [18]. Cases of diseases like diarrhea, conjunctivitis and dysentery are also increasing due to the extreme heat and potential variations in summer precipitation [2,14,18]. The additional burden of infectious diseases and conditions like mental anxiety, malnutrition, and heat stroke are also affected by climate change. The cumulative effect of all the projected changes in health and health-related factors may affect the human health of the country severely and may create huge burdens on the health system, beyond the capacity of the people and the country’s limited resources. Finally, it can severely weaken the ability of people to cope with their health concerns.

Many of the world’s developing countries have very weak health care systems. They also have insufficient resources to fulfill the very basic health needs of their people. Bangladesh is among one of these countries. Its total expenditure on health as a percentage of its GDP was 3.2 per cent in 2005 [19] and 3.4 per cent in 2007 [20]. Similarly, “per capita spending on health was Bangladesh Currency Taka (BDT) 391 ($9.2) in 1997, BDT 568 ($10.5) in 2001 and BDT 1,111 ($16.1, US$1 = Bangladesh currency taka (BDT) 74.00 in 2011) in 2007” [20]. In South Asia, Bangladesh had the lowest per capita expenditure on health in 2007—US$ 16.1 [21]. However, in comparison to other south Asian countries, private expenditure on health in Bangladesh is very high (74% in 2007) [20]. The lower level of the expenditure of GDP on health, higher level of out of pocket payments, and projected climate variability induced diseases will make the health system incapable of serving the need of the people at village level. Moreover, due to the high price of medicine, poor access to quality care, and unaffordability of modern medicine, options for coping with health problems for the poor people using modern medicine only are very limited [22]. This reality has encouraged people to seek alternative means (traditional medicine) to cope with their health problems.

The use of traditional medicine exists in every part of the world [23]. Among the user countries “the major areas are Chinese, Indian and European traditions” [24]. Use of traditional medicine made health system “pluralistic” in many countries of the world of the world, where forms of traditional medicines are changing in the diffusion of western medicine [25]. Traditional medicine has been conceptualized and perceived in various terms. World Health Organization has defined traditional medicine as “the sum total of the knowledge, skills, and practices based on the theories, beliefs, and experiences indigenous to different cultures, whether explicable or not, used in the maintenance of health as well as in the prevention, diagnosis, improvement or treatment of physical and mental illness” [23]. The terms “complementary medicine” or “alternative medicine” are used inter-changeably with traditional medicine in some countries. They refer to a broad set of health care practices that are not part of that country’s own tradition and have not been “integrated into the dominant health care system” [26,27]. The National Encyclopedia of Bangladesh has defined traditional medicine using similar characteristics [28]. Bangladesh national health accounts (BNHA) has coded traditional medicine as BP5.5 for unconventional/traditional practitioners and BP5.5.9 for other unconventional providers (except *Homeopathic, Ayurvedic and Unani*) [20]. Considering the use and importance of traditional medicine, WHO has officially adopted the use of traditional medicine in 1978 [29,30]. The use of traditional medicine has greater significance in healing sickness and disease for local people because of its long tradition, effective outcome and their long-held belief in its effectiveness.

Research on traditional medicine in Bangladesh has been conducted from different perspectives. In 1994, Blanchet studied the impact of rituals, customs and superstitions on pregnancy and child birth in rural Bangladesh. In her research, she described the influence of Muslim, Hindu, and *kobiraji* healers’ (the profession of an Ayurvedic physician—who recommends treatment based on herbal preparations, diet, yoga, and purification) treatment on the health of the expectant mothers in rural areas [31]. Another study discovered the use of indigenous solutions in modern hospitals in low income countries where people fight over limited resources [32]. A few other researchers have also carried out projects in rural areas to find out the religious, traditional and cultural practices used in healing different diseases [31,33-36]. These studies have shown how cultural, religious and customary practices have been used in healing health problems. In Bangladesh, Muslim religious leaders also expressed satisfaction, and a positive attitude towards the use of complementary and alternative medicines (CAM) [37].

Few studies have been conducted on the magnitude of the use of traditional medicine in rural areas. Little attention has been given to the contribution of traditional medicine on health-coping strategies in rural areas of Bangladesh. Little is known about the use of traditional medicine to cope with diseases sensitive to climate change –that is diseases linked climatic factors in particular temperature, precipitation, or humidity– among people vulnerable to climate change. Exploring this ignored area could help formulate or reform appropriate need-based adaptation measures for the health of the people in Bangladesh. The aim of the study is to describe the extent of the use of traditional medicines to cope with climate sensitive diseases in a resource poor setting. Specifically, ‘disease-specific’ use of the components of traditional medicines to avert climate sensitive health problems was presented.

## Methods

Use of traditional medicines as health coping strategies for the people vulnerable to climate change was studied using a ‘mixed methods research design’ as described in the literature [38-40]. Data were collected from two villages during September 2010 to March 2011: One village was located in the Rajshahi district, the northern part of Bangladesh, and the other village was in the Khulna district, located in the southern part or coastal belt [3]. Based on national data, the two study areas were chosen for their similarity in term of socio-demographic, climatic, occupational and livelihood patterns [41,42]. To comply with the research ethics, oral or written consent was taken from each respondent before interview. The Ethical Commission of Heidelberg University, Germany and the research evaluation committee of the Department of Population Sciences, University of Dhaka, Bangladesh approved the study.

Full details of the research tools that are summarized below were published in Haque et al. 2012 [14]. To ensure the validity and reliability of the instruments, we did a literature review to identify issues related to cultural, socio-economic aspects and use of traditional medicine, then consulted with anthropologists, sociologists and health experts in Bangladesh to select each of the items related to the use of traditional medicines as part of health-coping strategies, and finally were pretested the tools explicitly in the field. The data collection was conducted by the 3 female and 2 male interviewers who were also involved in developing the interview schedule, and such involvement deepened their understanding of the themes, concepts and questions. Considering the social context of the areas studied, the interviewers received necessary training in socio-cultural sensitivity, discretion concerning the confidentiality of the data collected, and rapport building with the interviewees as rural people. The researcher monitored the field work full-time to ensure the quality of the data.

More detail about the methods of the research was published in Haque et al. 2012 [14]. For the quantitative part of the study, we prepared individual lists of all the households in the two villages (1500 in Dhuroil village and 750 in Sachibunia village = 2,250 total) where 297 and 153 (total 450) households were interviewed, respectively. Households and number of respondents (male and female) from each village were selected using probability proportionate sampling to maintain proportionality. The male and female ratio of the respondents from each village were determined according to the national sex ratio (49 female per 51 male) [41]. No respondent dropped out the interview once it had started. The eldest male and female members were interviewed for the purpose of exploring in-depth information on the use of traditional medicine for coping with climate sensitive diseases and sickness. We assessed their opinion in a detailed section of the interview which included 76 questions regarding the use of traditional medicine in coping with climate sensitive diseases. The solicited responses were categorical (“Yes,” “No,” “Don’t Know,” “Not Applicable”). The Statistical Package for the Social Sciences (SPSS-17.0) was used to analyze the quantitative data.

Qualitative method of the study also included 12 focus group discussions (FGD), six with females and six with males, and 15 key informant interviews (KII). These FGDs and KIIs were conducted by the research team using a guideline on the use of traditional medicine. Interviews were recorded with the permission of the participants and played back to the respondents. FGD and KII included the same issues and items on traditional medicines which were used in the survey. A group of around ten people participated in each FGD. The participants of the FGDs and KIIs were senior community members, non-governmental organization officials, village doctors, local political leaders and teachers with a similar socio-demographic background and representative of the survey participants from the study areas. Transcribed FGDs and KIIs were analyzed and coded into broad themes (opinion regarding traditional medicine use, how they learned the use of TM, effectiveness of the use of TM) related to the use of traditional medicines for averting climate sensitive diseases. The broad themes were constructed in reference to the objectives of the research. The content analysis was performed manually.

## Results

In the quantitative study, 53% of the respondents were male and 47% were female. There was no significant difference among the educational backgrounds of the male and female respondents (Table 1). A major portion of the respondents (27%) had no formal education and 25 per cent had primary education only. The mean age of the respondents was 39.9 years with a standard deviation of 12.2 years. There was also no significant difference among the age groups of the male and female respondents. A significant difference in the occupations of the respondents was found relating to gender (Table 1). More information about socio-economic and demographic aspects of the respondents was presented elsewhere [14].

**Table 1:** Characteristics of the survey participants (n = 450), by sex

**Household characteristics of respondents**	**n (%)**	**n (%)**
	**Male**	**Female**
**Sex**	**238 (52.9)**	**212 (47.1)**
**Age (Years)**	Male	Female
Below 30	14.0	20.2
31-40	15.1	16.2
41-50	12.9	7.3
51-60	6.2	3.1
61+	4.7	0.2
**Mean ± Standard deviation**	**Mean for male = 42**	**Mean for female = 35**
	**Over all Mean = 39.9 years. S.D. = 12.2**	
Comparison male/female (chi-square)	χ = 7.56, p = 0.109	
Total 450	,	
**Education**	Male	Female
No Formal Education	13.8	13.1
Primary Education	13.3	11.6
Junior Secondary	8.4	12.5
Secondary School Certificate (SSC)	5.8	5.1
Higher Secondary (HSC)	5.1	2.9
Bachelor Level	4.5	1.1
Masters level	2.0	0.9
Comparison male/female (chi-square)	χ = 3.76, p = .709	
**Occupation (Total 450)**	Male	Female
Agricultural activities	30.0	1.1
Homemaker/Housewife	0.0	41.6
Services (Govt. NGO,)	8.2	2.4
Business (small and medium)	11.1	0.9
Others (village doctor, rickshaw-puller, fisherman)	3.6	1.1
Comparison male/female (chi-square)	χ = 81.57, p = .0001	
**Health Care Providers (village level)**		Count
Union Family Welfare Center		1
Satellite Clinic		1
Village doctor** (drug seller)		23
Paramedics		2

**People who has only a training of primary health care for about 15 days to 90 days.

Villagers in each selected village had access to one union family and health welfare center and one Upazila health complexes. There were also family welfare visitor/assistant and one satellite clinic. Road communication with these health facilities was good. These were the main public providers of modern medicines for the respondents/villagers. In addition, there were many small drugstores, village doctors and one paramedic in the villages, but there is no private chamber of the MBBS doctor.

Table 2 shows the% of positive responses for traditional methods used to treat very common and climate-sensitive diseases. Almost all respondents had used various kinds of traditional medicine in healing different climate sensitive diseases or sicknesses that their family members have had. Seventy six types of traditional medicine were used in healing 11 types of very common and climate-sensitive diseases (Table 2).

**Table 2:** Use of different Indigenous/Traditional/Alternative medicine in rural communities for healing different diseases and sickness

**Disease wise use of different Indigenous/Traditional/Alternative medicine**			
**Diarrhea**	**Yes (%)**	**Dengue**	**Yes (%)**
Drink saline at home	96.3	Use mosquito net all the times	65.0
Drink the water used to wash puffed rice (chira)	90.1	**Malaria**	
Drink the water of boiled rice	59.2	Use mosquito net all the times	75.9
Stop eating spicy food	73.4	**Skin Diseases**	
Increase the frequency of drinking water	30.9	Drink the juice of Margo’s leaf	66.3
Drink green coconut water	72.7	Take shower with boiled water of Margo leaves	81.4
Take rice soup	31.8	Take selective food	89.2
Eat thankuni (*Centella asiatica*) leaf	15.0	Avoid goods and stuffs of the affected person	87.6
Eat telakucha (*Coccinia grandis*) leaf	8.0	Rub chirota essence on body	11.6
Eat shiuly (Jasmine) leaf	2.0	Rub turmeric on body	20.5
Eat helencha (*Enhydra fluctuans*, Asteraceae) leaf	4.6	Shower with Alum mixed water	29.8
Drink juice	22.6	Rub the essence of Marigold flower on body	4.6
**Dysentery**		Rub mulberry (toot) leaf on body	2.3
Eat smashed green banana	79.1	**General clod/coughing/fever**	
Eat ripe banana	71.3	Use massage oil on body and head	91.9
Take a mixture of mango, banana and sugar	57.6	Use salted hot water to gargle	86.4
Drink goat milk	11.2	Pour water on head	97.5
Drink the water used to wash puffed rice (chira)	87.0	Take water vapour	59.0
Drink juice of thankuni leaf/Centella asiatica (L.)	24.9	Take honey	63.5
Drink juice of telakucha leaf/Coccinia grandis (L.) J.	11.6	Take sour fruits	64.0
Drink juice of pathorkuchi (Bryophyllums)leaf	1.5	Drink warm water	64.9
Drink juice of tulshi (Basil, Ocimum Sanctum) leaf	8.3	Drink juice of tulshi (basil) leaf	22.7
Drink juice of helencha leaf/(*Enhydra flactuans*)	6.5	Drink juice of basok (Adhatoda vasica)leaf	4.2
Drink juice of amboli leaf	8.3	Drink juice of sheuly (Jasmine) leaf	3.7
Drink juice of margo leaf	8.6	Take ginger/garlic with mustered oil	12.4
Drink juice of promigant leaf	6.5	Eat papaya	19.4
Drink juice of adaboron leaf	1.2	Eat pineapple	19.9
Eat hot jilapi (a fried of mixed wheat flower and sugar)	1.5	Eat pomegranate leaf	4.6
Eat curd	14.9	**Typhoid**	
Eat papaya	19.4	Drink much water	90.5
Eat custard apple	19.5	Pour water on head	93.2
		Eat mixture of rice, burley, sugar and lemon	14.3
**Asthma**	**Yes (%)**	**Headache**	**Yes (%)**
Try to keep neat and clean	95.2	Use local made balm	77.8
Try not to catch cold	96.2	Take head massage	91.3
Eat tulshi (Basil) leaf	13.8	Avoid sun heat/Stop going out in the sun	90.5
**Jaundice Hepatitis B**		Take much rest/sleep more	97.0
Take the stew of cat fish	39.4	Take ginger mixed tea	18.8
Take shaddock	48.2	**Prickles Blistering/Ghamachi**	
Drink sugarcane juice	95.7	Take shower in rain water	85.1
Drink green coconut water	89.2	Rub ice on the body	21.2
Avoid spicy food items	90.0	Take shower with Margo leaves mixed water	44.2
Take enough rest	91.5	Take shower with antiseptic mixed water	55.6
Drink orohor juice (Cajanus Cajan (L.) Millsp.) leaf	67.7	Use prickly-heat powder	96.5
Eat Papaya	29.4	Prick and take out the pus from it	89.6
Drink molasses/condensed sugar juice	26.2	Rub fitkiri (Alum) all over the body	18.0
Take green banana	20.6		
Drink raw milk	10.4		
Eat rice soup	16.0		
Eat helencha leaf/(*Enhydra flactuans*)	5.7		
Eat custard apple juice	10.3		
Take barley	4.7		

(n = 450).

FGDs and KIIs informed us that there has been a long tradition of the use of traditional medicines for healing different diseases and sickness. However, recently the uses of traditional medicines have increased as compared to five to ten years ago due to increased rates of climate sensitive sickness and diseases. They assumed that climate variability sensitive diseases had increased by 30–40 per cent compared to five to ten years back. They also explained that the use of modern medicine was within the practice of the respondents but the availability and also affordability of modern medicine were limited for the respondents. In contrast, they opined that the use of traditional medicine was within their known knowledge and practices; they could use it as and when needed, and it was effective and less expensive. A KII from Khulna district informed us that there were many people he knew of whom once used modern medicines but now rely on traditional medicine. They pointed out that increased frequency of climate induced diseases and sickness and poor availability, affordability as well as limited access to modern medicine compelled people to use traditional medicines to cope with adverse health situations.

The FGDs and KIIs added that there were no formal or written directories of the traditional medicine available to them. Most of the respondents reported that they *had learned about the use of traditional medicines via oral tradition and their application by their family members and elders. But there was no written record* of *the items used in traditional medicines for specific diseases/sicknesses. And there was no provision for any formal training of the family members for the preparation of the traditional medicine for specific diseases or sickness.* A FGD participant added*, “We get benefits from the use of traditional medicine and we use it. But still there are no researches or scientific findings on the effectiveness of the use of the items of traditional medicines”*. They did not get any prescription or suggestion from hospital or doctors about the use of traditional medicines. Responding users also did not consult with doctors about the use of traditional medicine even though they used it. Respondents were also used traditional medicine for both communicable and non-communicable diseases. They used traditional medicines at the beginning stages of sickness and simultaneously during the use of modern medicine. They also employed traditional medicines if they could not afford modern medicine, or if modern medicine was ineffective in healing diseases or sickness. The major types of diseases for which traditional medicines were used include: diarrhea/cholera, dysentery, asthma, jaundice, dengue, malaria, skin diseases, general cold/coughing/fever, typhoid, headaches, and prickles (heat rash) (Table 2). Eighteen traditional items were used to heal waterborne diseases such as dysentery; and 15 traditional medicines items were used for healing jaundice, general cold/coughing/fever. Irrespective of education, occupation, income and gender, all of the respondents used traditional medicine.

Respondents reported that diseases and sickness had increased due to changes in heat, cold and precipitation in the areas [14]. Coping with the increased frequencies of diseases was a major challenge for the respondents. A women FGD participant from Rajshahi district mentioned that she had experienced some sickness, which she associated with the changes in climate variability and her inability to cope with it. She said that “*disease, disease and diseases; all over the year [sic] at least one member of our family is sick due to climate variability. Treatment is not available at union clinics. So we try to manage diseases and sickness with our own experiences and knowledge [traditional medicines]”.* A female KII member from Khulna district queried that *“there is no treatment and medicines (modern) at clinics at our community hospital [sic]. So what can we do with these increased frequencies of sickness of the family members?”*

## Discussion

All the respondents were from agrarian based rural settings and had little formal education. However, they have a clear perception about the effect of climate change and variability on their health [14]. The majority of diseases/illness that have been reported to have increased because of changes in heat, cold and rainfall are diarrhea, dysentery, asthma, jaundice, dengue, malaria, fever, the common cold, headaches, stomachaches, psychological disorders, burning sensations, conjunctivitis, high blood pressure, sun-burns, skin- blisters, typhoid, pox, rheumatism and general bodily aching, pneumonia, measles, heatstroke, and malnutrition related disease [14]. In coping with this increased number of climate sensitive diseases/sickness, the respondents have reported that they rely on a variety of traditional and modern medicines [43]. Respondents reported that they had used traditional medicines to heal themselves and their family members. More than 76 types of traditional medicines were used for healing 11 of the most frequently suffered climate sensitive diseases. This indicates that respondents are actively coping with the adverse health situation with the means they have [44]. Similar use was observed in Western Nigeria where thirty one plants are commonly used by the traditional healers [45]. Respondent reported that traditional medicines were available, easily manageable, effective and affordable as found in Chinese and Taiwanese society [46,47]. Additionally, they have a cultural knowledge of how they should function. They use traditional medicines at the onset of the diseases/sickness. They use traditional medicines regardless of concomitant modern medicine use and and if the modern medicines appear ineffective. This implies that there is a consistent tendency towards using traditional medicines among the respondents and that they trust their effectiveness.

While the importance of traditional medicine has been studied in the US [48-51], New Zealand [52], India [53-55] and many other countries [56,57] there has been little attention paid by the researchers to the role of traditional medicines in Bangladesh. Findings also showed that there is no integration between the doctors and patient regarding the traditional medicines. This study offers the first such type of research conducted in Bangladesh where the extent of the use of traditional medicines have been studied as an essential strategy for coping with the increase of climate sensitive diseases and sickness of the communities vulnerable to climate change. The study also examines the use of various items of traditional medicines in treating a single disease among people of the study areas. The findings of the study offer information on how extensively community people have used traditional medicine as a strategy for coping with diseases. This study findings may be important in “stimulating the generation, translation, and dissemination of valuable knowledge”, as well as for promoting effective and efficient policy options for a country with limited resources [58,59]. The findings of the study will provide policymakers, donor agencies, non-government/international organizations and others with information necessary for formulating appropriate policy to cultivate the use of traditional medicine effectively for people vulnerable to climatic events in a resource-poor setting. The study findings provide end-line information to policymakers, which can be used to modify and improve Bangladesh’s health system, so that it aligns with the needs of the populations vulnerable to climate-change related health problems.

The use of indigenous knowledge and coping strategies is considered very important in formulating adaptation and mitigation strategies. United Nations Framework for Convention of Climate Change (UNFCCC) has developed a database to accumulate local coping strategies from different parts of the world. The database catalogues different hazards, impacts and local strategies by region [60]. However, there is no evidence collected or case studies of local coping strategies concerning health hazards. This study, therefore, may also be used to enrich the database of UNFCCC, providing local health coping information centered around using traditional medicine in a climate-vulnerable and resource-poor setting. The extensive use of traditional medicines for coping with sickness/diseases indicates that people are aware of adverse health problems and want to adapt to them using available, affordable and easily accessible indigenous solutions based on resources and knowledge they have [32,61]. The national health policy of Bangladesh has not taken the use of traditional medicines into consideration [62]. Health system policymakers and reform experts need to put effort into introduce accessible and affordable health systems for resource-poor settings by formally integrating the use of the traditional medicines [63]. This paper may provide policymakers with information and data on the rationale of integrating traditional medicines into a modern health care system of Bangladesh and countries with similar practices of using traditional medicine [29,64]. Such findings at the community level can also provide information to help prepare the National Adaptation Plan of Action’s (NAPA) priority list for addressing climate variability induced human health problems [65]. Banglapedia noted that an estimated 70 to 75 per cent people of the country use traditional medicine for their health care [28]. This research has found that 100 per cent of the village respondents have used and continue to use traditional medicine at any stages of their health coping strategies.

Many countries have already developed standard national classifications and terminologies for the use of traditional medicine [23,63], but Bangladesh has yet to develop a standard classification and repertoire of the terminologies of traditional medicines in Bangladesh. Also there are no directories indicating how many types of traditional means and items are used for treating any specific diseases or sickness in rural areas of Bangladesh.

The study was based on the collection of extensive qualitative and quantitative data but only from two villages, so the findings may be more applicable to an in-depth understanding of the use of the traditional medicine to cope with climate induced diseases. The generalization of these uses of traditional medicines to other areas of Bangladesh may require additional investigation. Additionally, there might be recall bias, as we also had to depend on the subjective judgments of the respondents’ experiences on the use of traditional medicine.

## Conclusion

The respondents of this study used traditional medicine irrespective of socio-economic or educational level. A systematic collection of such knowledge on the use of traditional medicines can help a climate vulnerable country initiate a directory of the components of traditional medicine used at community level for specific diseases and sicknesses. Such research findings may be useful for policymakers, researchers and development workers/partners in researching the effectiveness and importance of the use of traditional medicines as part of the health coping strategies of climate vulnerable people. It may also propel the policy makers to reformulate the existing health system policy of countries where people are struggling with limited resources. The findings of the study may urge WHO and national and international pharmaceutical companies to carry out research investigating the effectiveness of traditional medicines. Findings can provide information for public health researchers and policymakers to think about the possibilities of the integration of the traditional medicines with modern medicine. Empirically sound research into the components of traditional medicine which could be beneficially integrated with modern medicine to increase the ability of vulnerable, low-resource populations to cope with their climate-induced health risks would be advantageous at the community, national and international level.

### Ethical approval

Ethical approval was obtained from the Ethical Commission of Heidelberg University, Germany. The study was also approved by the research evaluation committee of the Department of Population Sciences, University of Dhaka, Bangladesh.

## Abbreviations

WHO: World Health Organization; GDP: Gross domestic product; BNHA: Bangladesh National Health Accounts; FGD: Focus group discussion; KII: Key informant interview; NAPA: National Adaptation Program of Actions; BCCSAP: Bangladesh Climate Change Strategy and Action Plan; BDT: Bangladesh Taka; USD: United States Dollar; UNFCCC: United Nations Framework for Convention of Climate Change; UNFPA: United Nation’s Fund for Population; EMMA: Erasmus Mundus Mobility in Asia.

## Competing interests

The authors declare that they have no competing interests.

## Authors’ contributions

MAH designed the study, developed the questionnaire, supervised the data collection, analyzed the data and wrote the paper. VL and RP contributed to the interpretation of the findings and writing of the manuscript. RS contributed to the development of the overall study concept, design of the study and drafting of the paper. All authors read and approved the final manuscript.

## Pre-publication history

The pre-publication history for this paper can be accessed here:

http://www.biomedcentral.com/1471-2458/14/202/prepub

## References

[B1] Germanwatch Global Climate Risk Index 2009. Weather-Related Loss Events and Their Impacts on Countries in 2007 and in A Long-Term Comparison 2007 Bonn: Germanwatch e.V.

[B2] Forests MoEa National Adaption Program of Action 2005 Dhaka: Ministry of Environment and Forests. Government of the People’s Republic of Bangladesh 1 63

[B3] CCC Climate Change and Health Impacts in Bangladesh 2009 Dhaka: Ministry of Forenst and Environment 1 82

[B4] IPCC Climate Change: Impacts, Adaptation, and Vulnerability. Contribution of Working Group II to the Third Assessment Report of the Intergovernmental Panel on Climate Change 2001 New York: Cambridge University Press 1032

[B5] McMichael AJ Butler CD Climate change and human health: recognising the really inconvenient truth Med J Aust 2009 191 11–12 595 596 20028274 10.5694/j.1326-5377.2009.tb03342.x

[B6] Global Environmental Change and Human Health Science Plan and Implementation Strategy Earth System Science Partnership (DIVERSITAS, IGBP, IHDP, and WCRP) Report No.4; Global Environmental Change and Human Health Report No.1 2007 (Please see online http://www.gechh.unu.edu/FINAL_GECHH_SP_UPDATED.pdf)

[B7] WHO Climate Change and Human Health - Risks and Responses. World Health Organization 2003 Geneva: WHO

[B8] Andy H Commentary: sustainable policies to improve health and prevent climate change Social Science & Medicine 2012 74 5 680 683 10.1016/j.socscimed.2011.12.008 22197291

[B9] WHO Gender, Climate Change and Health. World Health Organization 2009 Geneva: WHO

[B10] Preet R Nilsson M Schumann B Evengård B Global Health Action The Gender Perspective in Climate Change and Global Health Vol. 3 2010 North America Available at: http://www.globalhealthaction.net/index.php/gha/article/view/5720 10.3402/gha.v3i0.5720 PMC3001868 21160554

[B11] Address at the WHO congress on traditional medicine in 2008 by the Dr Margaret Chan, Director-General of the World Health Organization http://www.who.int/dg/speeches/2008/20081107/en/index.html#

[B12] Global estimates of burden of disease caused by the environment and occupational risks http://www.who.int/quantifying_ehimpacts/global/globclimate/en/index.html

[B13] Lokuge KM Smith W Caldwell B Dear K Milton AH The effect of arsenic mitigation interventions on disease burden in Bangladesh Environ Health Perspect 2004 112 11 1172 1177 10.1289/ehp.6866 15289162 PMC1247477

[B14] Haque MA Yamamoto SS Malik AA Sauerborn R Households’ perception of climate change and human health risks: a community perspective Environ Health 2012 11 1 1 10.1186/1476-069X-11-1 22236490 PMC3311088

[B15] Guha-Sapir D Hargitt D Hoyois P Louvain U Disasters WHOCRE Consortium P Oceanic USN Program AACI Assistance USAIDOUSFD Thirty Years of Natural Disasters 1974–2003: The Numbers 2004 Brussels: University of Louvain Presses

[B16] ADB Bangladesh: 2004 Flood, Response, Damage and Recovery Needs 2004 Dhaka: Asian Development Bank (ADB)

[B17] Bank W World Bank Bangladesh: Climate Change & Sustainable Development, Report Prepared by South Asia Development Teamvol. 21104 BD 2000 Dhaka: World Bank

[B18] Rahman A Climate change and its impact on health in Bangladesh Regional Health Forum 2008 2 1 16 26

[B19] World Health Organization Global expenditure database http://apps.who.int/nha/database/StandardReport.aspx?ID=REP_WEB_MINI_TEMPLATE_WEB_VERSION&COUNTRYKEY=84674

[B20] MOHAF Bangladesh National Health Accounts 1997–2007 2007 Dhaka: Ministry of Health and Family Welfare (MOHFW) 1 107

[B21] National Health Accounts Bangladesh http://www.who.int/nha/country/bgd/en/

[B22] Kaztman R United Nations Economic Commission for Latin America and the Caribbean. CEPAL Montevideo Activos y estructuras de oportunidades: estudios sobre las raíces de la vulnerabilidad social en Uruguay, 1a. edn 1999 Montevideo, Uruguay: Comisión Económica para América Latina y el Caribe, Oficina de Montevideo

[B23] WHO To define information standards for traditional medicine http://www.who.int/mediacentre/news/notes/2010/trad_medicine_20101207/en/

[B24] H.G V Similarities between various systems of traditional medicine. Considerations for the future of ethnopharmacology J Ethnopharmacol 1991 35 2 179 190 10.1016/0378-8741(91)90071-K 1809825

[B25] Lock M Medicine in Chinese Cultures: Comparative Studies of Health Care in Chinese and Other Societies. Arthur Kleinman, Peter Kunstadter, E. Russell Alexander, and James L. Gale Medical Anthropology Newsletter 1978 9 4 11 13 10.1525/maq.1978.9.4.02a00140

[B26] S B Kris H WHO Definition of Health, Rethinking the International Encyclopedia of Public Health 2008 Oxford: Academic Press 590 597

[B27] Field J Social Capital 2003 London; New York: Routledge

[B28] Banglapedia: National Encyclopedia of Bangladesh Traditional Medicine 2006 Dhaka: Asiatic Society of Bangladesh

[B29] Chunhuei C Integrating traditional medicine into modern health care systems: examining the role of Chinese medicine in Taiwan Soc Sci Med 1994 39 3 307 321 10.1016/0277-9536(94)90127-9 7939847

[B30] Volken-Reinert T Elemente des Vertrauens : Internetdiffusion in den Transformationsländern als Paradigmatest 2002 Bern; New York: P. Lang

[B31] Theresa B Meanings and Ritiuals of Birth in Rural Bangladesh 1984 Dhaka: The University Press Limited

[B32] Shahaduz Z Poverty and violence, frustration and inventiveness: hospital ward life in Bangladesh Soc Sci Med 2004 59 10 2025 2036 10.1016/j.socscimed.2004.03.007 15351470

[B33] Ellickson J A Believer Among Believers: The Religious Beliefs Practices and Meanings in a Village in Bangladesh 1972 Michigan: Michigan State University

[B34] Islam M Folk Medicine and Rural Women in Bangladesh 1980 Dhaka: Women for Women

[B35] Islam M Women Health and Culture: A Study of Beliefs and Practices Connected with Female Diseases in a Bangladesh Village 1985 Dhaka: Women for Women

[B36] Rk H Text Book of Community Medicine and Public Health 1992 Dhaka: RKH Publishers

[B37] Vargas Cetina G Palomo Infante MD De lo privado a lo público : organizaciones en Chiapas, 1. edn 2002 México: CIESAS Centro de Investigaciones y Estudios Superiores en Antropología Social : Miguel Angel Porrúa Grupo Editorial

[B38] Morse MJ Niehaus L Mixed Method Design: Principles and Procedures (Developing Qualitative Inquiry) 2009 Walnut Creek, California: Left Coast Press

[B39] Creswell WJ Research Design: Qualitative, Quantitative, and Mixed Methods Approaches, vol. Third 2009 New Delhi: Sage Publications, Inc

[B40] Creswell WJ Qualitative Inquiry & Research Design: Choosing Among Five Approaches, vol. 2nd 2007 New Delhi: Sage Publications, Inc.

[B41] BBS Population Census Report 2011 2011 Dhaka: Ministry of Planning. Government of the People’s Republic of Bangladesh

[B42] National Institute of Population R, Training, Mitra, Associates, and Macro I Bangladesh Demographic and Health Survey 2007 2009 Dhaka: NIPORT, Bangladesh and Calverton, Maryland, USA

[B43] UNDP Perception of Illness and Health Seeking Behaviur Among Five Ethnic Groups (Belgali, Chakma, Marma, Tanchangya and Tripura) in the Chittagong Hill Tracts 2010 Dhaka: UNPD

[B44] Kleinman A Program JEFICASHSGHS Washington U Medicine in Chinese cultures: comparative studies of health care in Chinese and other societies 1976 Washington: National Institutes of Health

[B45] Abo KA Fred-Jaiyesimi AA Jaiyesimi AEA Ethnobotanical studies of medicinal plants used in the management of diabetes mellitus in South Western Nigeria J Ethnopharmacol 2008 115 1 67 71 10.1016/j.jep.2007.09.005 17950547

[B46] Kleinman A Sciences JEFICASH Washington U Medicine in Chinese Cultures: Comparative Studies of Health Care in Chinese and Other Societies : Papers and Discussions from a Conference Held in Seattle, Washington, U.S.A., February 1974 1976 Washington: U.S Department of Health, Education, and Welfare, Public Health Services, National Institutes of Health, John E. Fogarty International Center for Advanced Study in the Health Sciences, [International Cooperation and Geographic Studies Branch]

[B47] Kleinman A Patients and Healers in the Context of Culture: An Exploration of the Borderland Between Anthropology, Medicine, and Psychiatry 1980 Los Angeles: University of California Press

[B48] Morrison AR Ellsberg M Bott S World Bank Addressing Gender-Based Violence in the Latin American and Caribbean Region a Critical Review of Interventions Policy Research Working Paper 3438 2004 Washington, D.C: World Bank

[B49] Nedelchev D Sot︠s︡ialen kapital i ikonomichesko razvitie, 1. izd. edn 2004 Sofii︠a︡: Akademichno izdatelstvo “Marin Drinov”

[B50] Gastaldi F Milanesi E Barca F Capitale sociale e territorio: risorse per l’azione locale 2003 Milano, Italy: FrancoAngeli

[B51] Community Action Council for Lexington-Fayette Bourbon Harrison and Nicholas Counties., National Civic League (U.S.), J. Thomé Community Development Consulting Understanding & Measuring Social Capital: Featuring the Winburn Neighborhood, Lexington, Kentucky 2001 Lexington, KY: Community Action Council for Lexington-Fayette, Bourbon, Harrison, and Nicholas Counties

[B52] La Rosa Huertas L, Ford Foundation Políticas de promoción de la salud y capital social 2002 Perú: s.n

[B53] Gambino F Mingione E Pristinger F Distanze e legami : una ricerca su capitale sociale e diseguaglianze nel Veneto, 1a edn 2003 Roma: Carocci

[B54] Kawade M Bessho S Katō R Kōreika shakai ni okeru shakai shihon : bumonbetsu shakai shihon o kōryoshita chōki suikei 2003 Tokyo, Japan: Naikakufu Keizai Shakai Sōgō Kenkyūjo

[B55] Hooghe M Sociaal kapitaal in Vlaanderen : verenigingen en democratische politieke cultuur 2003 Amsterdam: Amsterdam University Press

[B56] Szczepański MS Rojek P Wyższa Szkoła Zarządzania i Nauk Społecznych w Tychach Cnoty i instytucje obywatelskie w społeczności lokalnej 2001 Tychy: Śląskie Wydawnictwa Naukowe, Wyższa Szkoła Zarządzania i Nauk Społecznych w Tychach

[B57] Evers A Rauch U Stitz U Von öffentlichen Einrichtungen zu sozialen Unternehmen: hybride Organisationsformen im Bereich sozialer Dienstleistungen 2002 Berlin: Edition Sigma

[B58] WHO The World Health Report 2012: No Health Without Research 2012 Jeneva

[B59] WHO WHO The World Health Assembly Resolution 58.34 2005 Jenava: WHO

[B60] United Nations Framework Convention on Climate Change (UNFCCC) Local coping stratigies database http://maindb.unfccc.int/public/adaptation/

[B61] Robinson D Victoria University of Wellington. Institute of Policy Studies Building Social Capital 2002 Wellington, N.Z: Institute of Policy Studies, Victoria University of Wellington

[B62] Health popicy of Bangladesh http://nasmis.dghs.gov.bd/mohfw/index.php?option=com_content&task=view&id=388&Itemid=483

[B63] Knack SF Washington DC World Bank. Development Research Group. Regulation and Competition Policy Social Capital and the Quality of Government Evidence from the U.S Policy Research Working Paper 2504 2000 World Bank, Development Research Group, Regulation and Competition Policy

[B64] François P Social Capital and Economic Development 2002 London; New York: Routledge

[B65] Forests MoEa National Adaption Programme of Action (NAPA) 2009 Dhaka 1 78

